# Systemic autoimmune diseases and work outcomes in Brazil: a scoping review

**DOI:** 10.11606/s1518-8787.2022056003918

**Published:** 2022-04-11

**Authors:** Rafael Alves Cordeiro, Frida Marina Fischer, Samuel Katsuyuki Shinjo

**Affiliations:** I Universidade de Sao Paulo Faculdade de Medicina Departamento de Reumatologia São Paulo SP Brasil Universidade de Sao Paulo. Faculdade de Medicina. Departamento de Reumatologia. São Paulo, SP, Brasil; II Universidade de Sao Paulo Faculdade de Saude Publica Departamento de Saude Ambiental São Paulo SP Brasil Universidade de Sao Paulo. Faculdade de Saude Publica. Departamento de Saude Ambiental. São Paulo, SP, Brasil

**Keywords:** Autoimmune Diseases, Absenteeism, Employee Performance Appraisal, Occupational Health, Review

## Abstract

**OBJECTIVE:**

To review articles that assessed work-related outcomes such as workability, work productivity, presenteeism, absenteeism, sick leave, return to work, and employment status of Brazilian patients with rheumatoid arthritis, systemic lupus erythematosus, systemic sclerosis, Sjögren’s syndrome, and systemic autoimmune myopathies.

**METHODS:**

This study was conducted in Medline databases (PubMed), SciELO, and Lilacs through a combination of descriptors of interest. Studies published until December 2020 were considered in the search strategy.

**RESULTS:**

Eight out of 90 articles met the eligibility criteria and were included in this review. The studies are highly heterogeneous. Most of them are cross-sectional, and all of them address rheumatoid arthritis or systemic lupus erythematosus. A common denominator among these studies is the high proportion of patients outside the labor market.

**CONCLUSIONS:**

In general, the studies show unfavorable labor outcomes and impaired participation in the Brazilian workforce among the samples of patients assessed. There is a need to better understand several topics about Brazilian patients with systemic autoimmune diseases and their work context, as well as to conduct studies focusing on rarer diseases and on the themes of return and reintegration to work.

## INTRODUCTION

Systemic autoimmune diseases are a broad range of conditions characterized by dysfunction of the immune system, resulting in multi-tissue inflammation and damage^1^. Among these diseases, we find rheumatoid arthritis, systemic lupus erythematosus, systemic sclerosis, Sjögren’s syndrome, and systemic autoimmune myopathies.

Reduced work capacity, presenteeism, absenteeism, and sick leave are frequent outcomes among individuals with rheumatic diseases^2^. In general, work disability results from psychological and socioeconomic factors, as well as from the severity of the disease and damage accumulation related to inflammatory activity and treatment. If demands at work are greater than an individual’s physical and mental adaptation resources, workability tends to worsen, leading to presenteeism, absenteeism, and early exit from the labor market^3,4^.

The impact of sick leave on social security is a global concern. Prolonged absences from work, especially those related to mental and musculoskeletal disorders, generate considerable economic and human costs for the social security system and for society^5,6^. In general, the longer the period away from work, the more difficult and challenging the return and reintegration to work. For this reason, it is essential to gain a better understanding of the factors associated with unfavorable work outcomes in order to develop strategies that aim to reduce the socioeconomic burden of specific chronic diseases^5,6^.

To the best of our knowledge, no scoping reviews have addressed work-related outcomes in patients with systemic autoimmune diseases in Brazil. Thus, this study aimed to review articles that assessed variables, such as workability, work productivity, presenteeism, absenteeism, sick leave, return to work, and employment status in patients with specific autoimmune rheumatic disorders in Brazil.

## METHODS

Eligibility criteria were observational studies that included Brazilian patients diagnosed with rheumatoid arthritis, systemic lupus erythematosus, systemic sclerosis, Sjögren’s syndrome, or idiopathic inflammatory myopathies, and that evaluated work-related outcomes for these patients. No language restrictions for inclusion were considered. Studies were conducted until December 2020.

Databases and search strategy: the search for articles was carried out in Medline (via PubMed), Scientific Electronic Library Online (SciELO), and Latin America and Caribbean Health Sciences Literature (Lilacs). The search strategy for Medline (via PubMed) used a combination of Medical Subject Headings (MeSH), as follows: (Connective Tissue Diseases OR Autoimmune Diseases OR Rheumatic Diseases OR Arthritis, Rheumatoid OR Lupus Erythematosus, Systemic OR Scleroderma, Systemic OR Scleroderma, Limited OR Scleroderma, Diffuse OR Sjogren’s Syndrome OR Myositis OR Polymyositis OR Dermatomyositis) AND (Return to Work OR Work Capacity Evaluation OR Work Performance OR Sick Leave OR Absenteeism OR Presenteeism OR Employment OR Unemployment) AND (Brazil OR Latin America). This strategy was adapted to the other databases.

Selection of articles and data extraction: the studies were selected in two steps. The first step involved screening studies based on titles and abstracts. For the following evaluation, the full texts of the selected articles were retrieved. Information was recorded with the author’s name, year of publication, country/region, study design, disease studied, disease characteristics, sample and patient characteristics, source of patients/recruitment strategy, work outcomes assessed, and work-related findings.

## RESULTS

We obtained 90 articles from the electronic search. Twenty-one duplicate articles were excluded. After the evaluation of titles and abstracts, 10 articles were selected for full-text evaluation. Among these, two studies were excluded, one because the population evaluated was composed of French patients^7^ and the other because it did not provide labor data of interest^8^.

In the end, eight articles met the eligibility criteria and were included in this review (Figure ).

**Figure:**
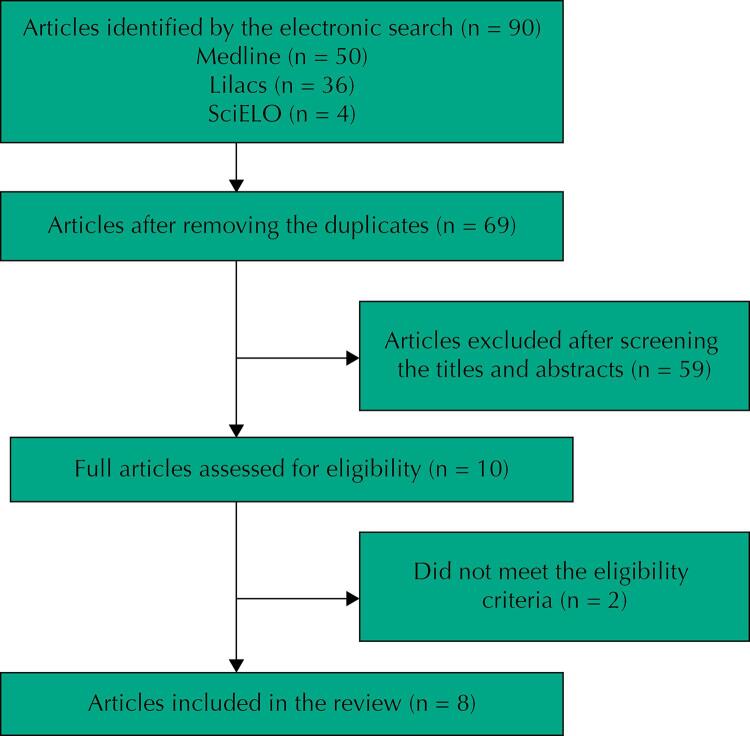
Flowchart of article selection.

The studies are highly heterogeneous, which substantially limits the comparison among them. We observe important differences in the analyzed populations regarding patient characteristics, sources of patients, ways to characterize disease severity, and results.

Most studies were cross-sectional. In six studies, the autoimmune rheumatic disease assessed was rheumatoid arthritis^9^. The remaining two studies evaluated systemic lupus erythematosus^15,16^. None of the eight articles addressed work outcomes in Brazilian patients with systemic sclerosis, Sjögren’s syndrome, or idiopathic inflammatory myopathy. Only three studies^10^ presented work-related outcomes other than employment statuses, such as work productivity, work impairment, presenteeism, absenteeism, and time on sick leave. None of the studies addressed the theme “return to work”.

Boxes 1 and 2 present the main features of the studies.

**Box 1:** Overview of descriptive characteristics, diagnosis, and sample specifications of the included studies.

First author	Year of publication	Study design	Country/region	Disease studied	Source of patients/recruitment strategy	Sample size and characteristics	Disease characteristics
Sacilotto et al.^9^	2020	Cross-sectional analysis (initial assessment of a cohort study)	Brazil/different geographic regions of Brazil	Rheumatoid arthritis	11 public reference centers	1115 RA patients; mean age (56.7); female (90%)	Mean disease duration: 12.7 years; median DAS28^a^: 3.5; median CDAI^b^ score: 9
Xavier et al.^10^	2019	Longitudinal	Latin America (Brazil, Argentina, Colombia, and Mexico)	Rheumatoid arthritis	18 rheumatology public and private clinics (4 Latin American countries)	68 RA patients in the Brazilian sample; mean age (45.9); female (86.8%)	Mean disease duration at baseline: 10.8 years; RAPID3^c^ score: remission (4.4%), low (1.5%), moderate (33.8%), high (55.9%)
Gomes et al.^11^	2018	Cross-sectional	Brazil/Blumenau (SC)	Rheumatoid arthritis	Primary care centers, specialty outpatient clinics, and pharmacy	296 RA patients included; 185 in the final sample; mean age (54.5); female (82.7%)	Mean disease duration: 127.8 months
Pinheiro et al.^12^	2013	Cross-sectional	Brazil	Rheumatoid arthritis	not mentioned	526 RA medical records; mean age (51.0); female (81%)	Mean disease duration: 6.5 years; physician’s subjective rating on disease severity: the majority were mild or moderate
Azevedo et al.^13^	2008	Cross-sectional	Brazil/São Paulo (SP)	Rheumatoid arthritis	Rheumatology outpatient clinics of a public hospital	192 RA patients; mean age (47.3); female (85.9%)	Mean disease duration: 9.8 years; ACR functional class I (13.5%), II (29.7%), III (56.3%), IV (0.5%)
Louzada-Junior et al.^14^	2007	Cross-sectional	Brazil/State of São Paulo	Rheumatoid arthritis	Department of Rheumatology (four university hospitals)	Occupational data available in 650 revised RA medical records; mean age (53.7); female (86%)	Mean disease duration: 7.8 years; ACR functional class I (50%), II (37%), III (11%), IV (2%)
Teixeira et al.^15^	2017	Cross-sectional	Brazil/Maceió (AL) and São Paulo (SP)	Systemic Lupus Erythematosus	3 university hospitals and private clinics	523 SLE patients; mean age (37.8); female (96%)	Mean disease duration: 9.6 years; mean ACR/SLICC-DI^d^ score: 1.4; mean Mexican-SLEDAI: 1.82
Appenzeller et al.^16^	2009	Longitudinal	Brazil/Campinas (SP)	Systemic Lupus Erythematosus	Rheumatology unit of a public university	167 SLE patients at study entry; mean age (32.1); female (94.6%)	Mean disease duration at study entry: 64.5 months; mean SLEDAI^e^: 11.5; 47.3% with SLEDAI ≥ 8

^a^ DAS28: Disease Activity Score 28-joints; ^b^ CDAI: Clinical Disease Activity Index; ^c^ RAPID3: Routine Assessment of Patient Index Data 3; ^d^ SLICC/ACR-DI score: Systemic Lupus International Collaborating Clinics/American College of Rheumatology Damage Index score; ^e^ SLEDAI: Systemic Lupus Erythematosus Disease Activity Index.

**Box 2:** Summary of employment status and other work-related findings reported in the studies on patients with systemic autoimmune rheumatic diseases in Brazil.

First author	Work outcomes assessed	How work outcomes were assessed	Work-related findings
Sacilotto et al.^9^	Employment status	Individual questionnaire; medical records used as secondary sources	Retired: 39%, housewives: 20%, disease support: 9%, unemployed: 5%, unemployed looking for work: 1%, formally registered worker: 16%, unregistered worker: 6%, self-employed worker: 4%. Employed patients: lower HAQ-DI^a^ averages and fewer erosions. Among the patients who retired: 56% due to RA; among the patients on disability benefit or temporarily retired: 82% due to RA.
Xavier et al.^10^	Wok productivity, absenteeism, presenteeism, and employment rate	WALS^b^, WPAI^c^, and WLQ-25^d^ questionnaires	Brazilian sample: WALS-score (0–36): 10.6 ± 6.8. WPAI-RA (0–100%): presenteeism (32,6%), absenteeism (5.8%), overall work impairment (5.9%). WLQ-25 - 4 dimensions (0–100%): work impairment (WI) due to physical demands (37.7%); WI due to time demands (29.3%); WI due to output demands (18.1%); WI due to mental-interpersonal demands (15.2%); WLQ-25 index: 5.9. The employment rate (Brazilian sample): 44.2% of the respondents of WPAI-RA. Considering the total sample (Brazil and other Latin American countries), higher educational levels were associated with better WLQ-25; previous orthopedic surgeries reduced absenteeism and work limitations; and worsening in disease activity was associated with decreased work productivity.
Gomes et al.^11^	Employment status	Self-reported employment status during structured interviews	Prevalence of RA working patients: 44.3%. The prevalence of working patients was 90% higher among those under 60 years, 132% higher among low-income individuals (< 2 minimum wages) and 73% higher among patients with high functional disability assessed through the HAQ^a^.
Pinheiro et al.^12^	Employment status, impairment while at work, and overall work impairment	Patient record form, patient self-completion form, and WPAI	Employed: 29%, retired: 24%, unemployed due to arthritis: 2% (other categories: student, self-employed, homemaker). Work and activity impairment rose with increasing disease severity. Severe RA patients had more unemployment due to arthritis and greater disease duration.
Azevedo et al.^13^	Employment status, days absent from work, time on sick leave, and time on retirement due to RA	Systematically structured interview	Early retirement due to RA: 24.5%, sick leave due to RA: 32.3%, working: 43.2%. Retired patients and those on sick leave tended to belong to lower socioeconomic categories, while those who were working were in the higher classes. Meantime on disability benefit: 2.25 years; meantime on retirement: 7.33 years.
Louzada-Junior et al.^14^	Employment status	Review of medical records	Formally employed: 31%; housewives: 40%; sick leave: 9%; unemployed: 4%; retired: 16%.
Teixeira et al.^15^	Employment status	Sociodemographic data were collected through a specific questionnaire	Unemployment rate: 63.7%. Among them, 47.4% were receiving social security and 23% referred to be permanently out of work because of SLE (higher for non-white patients). Out-of-work profile was associated with damage.
Appenzeller et al.^16^	Employment status	Structured interview at study entry and every 6 months for 3 years	At study entry, 70.7% of the patients were employed. After 3 years, 51.7% who were working at study entry, were still employed. Unemployment was predicted by the number of cognitive domains impaired, depression, fewer years of education, and the presence of anticardiolipin antibodies at study entry.

^a^ HAQ-DI: Health Assessment Questionnaire Disability Index; ^b^ WALS: Work Activity Limitation Scale; ^c^ WPAI: Work Productivity and Activity Impairment; ^d^ WLQ-25: 25-Item Work Limitations Questionnaire.

## DISCUSSION

This scoping review mapped the state of the art of work-related outcomes concerning systemic autoimmune diseases in Brazil. The point of convergence between the studies is the high prevalence of patients with autoimmune diseases outside the labor market. Furthermore, this review found important gaps in the literature, such as the scarcity of longitudinal studies evaluating work-related outcomes, as well as the lack of studies on return to work and labor outcomes for rarer autoimmune diseases.

The search strategy of this review contemplated five systemic autoimmune diseases (rheumatoid arthritis, systemic lupus, systemic sclerosis, primary Sjögren’s syndrome, and idiopathic inflammatory myopathies), as well as the main work-related outcomes (workability, absenteeism, presenteeism, sick leave, return to work, employment, and unemployment), focusing on Brazilian patients. Despite the broad scope of the research, we observe a marked predominance of rheumatoid arthritis among the articles included in this review. Surprisingly, none of the studies reported work-related outcomes for systemic sclerosis, Sjögren’s syndrome, or idiopathic inflammatory myopathies in the Brazilian population.

Workability is a multifactorial phenomenon that can be defined as the ability of a worker to manage work tasks, considering individual factors (e.g., physical and mental resources), work demands, and factors external to professional life. Workability is also influenced by worker competence and motivation, supervisor attitudes, ergonomics in the workplace, autonomy, control at work, environmental factors, and social circumstances^3,4^.

Reduced workability resulting in presenteeism, and absenteeism seems to be a frequent occurrence among individuals with different rheumatic diseases who remain employed. Presenteeism is the term used to describe the situation in which people continue going to work even with a poorly controlled health problem (physical or mental)^17^. Thus, attention has been paid to presenteeism as a potential predictor of absenteeism and disability pensions to assist in the early identification of people at risk of becoming unable to perform work activities^18^.

Assessing presenteeism is not an easy task, but it represents a relevant outcome for patients with chronic diseases since it may reflect the challenges and difficulties they face in maintaining performance despite health problems. Among the eight studies presented in Box 1, two included the evaluation of presenteeism^10,12^. Xavier et al.^10^ conducted the most comprehensive assessment of presenteeism and limitations at work among the studies encompassed in this review. The authors applied the questionnaires Workplace Activity Limitations Scale (WALS)^19^, Work Limitations Questionnaire (WLQ-25)^20^, and Work Productivity and Activity Impairment (WPAI)^21^ to assess the burden of rheumatoid arthritis on patients’ productivity.

The WALS is a feasible tool that can estimate the impact of the disease during the performance of activities in the workplace, including difficulties with the functioning of upper and lower limbs, concentration at work, pace, and scheduling of work. It can be expressed as a summed total score, ranging from 0 to 36 for the 12-item version, in which higher scores represent greater limitations in workplace activities^19,22^. The WLQ-25 measures the impact of chronic health conditions at work, with a focus on assessing disabilities while performing specific job demands. It consists of 25 questions organized into four subscales: time management, physical demand, mental-interpersonal demands, and output demands. The scores for each domain range from 0 to 100, with higher points indicating more limitations. It is also possible to estimate the WLQ Index using a weighted formula (considering all subscales)^20,22^. Besides estimating presenteeism, the WPAI questionnaire is applied to assess the effect of general health or a specific disease on absenteeism, and non-work activities. There are six non-summative questions related to a 7-day recall period that enable the estimation of four outcomes, ranging from 0 to 100%: absenteeism, presenteeism, overall work impairment due to health, and non-work activity impairment due to health. Higher scores indicate worse results^21,22^.

According to Xavier et al.^10^, the domain “physical demands” of the WLQ-25 was the most affected among patients with rheumatoid arthritis. Moreover, based on the longitudinal analysis, the worsening of disease activity had a negative impact on work productivity (estimated by the WALS) and on the WPAI domains “presenteeism” and “impairment of regular daily activities”^10^.

Absenteeism was assessed in two articles, both addressing rheumatoid arthritis^10,13^. Considering a recall period of seven days, Xavier et al.^10^ observed a 5.8% rate of absenteeism among patients with rheumatoid arthritis in the Brazilian sample, whereas Azevedo et al.^13^ reported the following average absenteeism from work due to rheumatoid arthritis: 1.31 and 14.6 days in the previous month and year, respectively.

Employment status, retirement, and being on sick leave were other work outcomes that differed considerably among studies focusing on rheumatoid arthritis. Some articles considered the formally registered workers, whereas others entailed a larger group, called the “working population,” which might have included unregistered and self-employed workers. Pinheiro et al.^12^ found that 29% of patients with rheumatoid arthritis were employed in their sample. Patients with severe rheumatoid arthritis and longer disease duration faced more unemployment compared with those experiencing mild/moderate disease^12^. Azevedo et al.^13^ observed that 43.2% of their sample of patients with rheumatoid arthritis was working at the time of the interview, and they tended to belong to higher income classes when compared with those who were retired or on sick leave. Conversely, even though the prevalence of working patients with rheumatoid arthritis was similar to that noted by Azevedo et al.^13^, in Gomes et al.^11^ samples, individuals with lower income and greater functional disability remained more frequently in the labor market^11^. The authors hypothesized that these findings might be related to the lack of information on disability retirement, lack of payment of social security contributions, and the need to work to avoid income loss in that rheumatoid arthritis population^11^.

In a large multiethnic sample of Brazilian patients with systemic lupus erythematosus, 63.7% were out of the labor market. Of these, 47.4% were receiving social security benefits and 23% reported being permanently out of work because of systemic lupus erythematosus^15^. The damage estimated by the American College of Rheumatology/Systemic Lupus International Collaborating Clinics Damage Index (SDI) had a significant influence on the risk of being unemployed^15^. The fact that working patients had a lower damage score than inactive ones suggests that limiting damage accumulation in these patients could optimize work-related outcomes.

Appenzeller et al.^16^ studied the impact of cognitive impairment on work status in patients with systemic lupus erythematosus. At baseline, they observed that unemployment was associated with the number of impaired cognitive domains (complex attention, memory, and executive functions), depression, fewer years of education, and the presence of anticardiolipin antibodies. After three years of follow-up, the unemployment rate was predicted by the impaired cognitive domain “reasoning and problem solving” in conjunction with the factors previously mentioned. Due to the fact that a high proportion of patients with systemic lupus erythematosus do not spontaneously report cognitive impairment and considering that it is not routinely screened, the authors suggest that an appropriate screening tool for cognitive impairment would be useful in clinical practice^16^.

In conclusion, although highly heterogeneous and challenging to compare, the studies covered in this review converge on the significant impact of systemic autoimmune rheumatic diseases, particularly rheumatoid arthritis and systemic lupus erythematosus, on the participation of patients in the Brazilian workforce. Despite the extensive literature search, a knowledge gap was evident on several relevant topics about Brazilian autoimmune rheumatic patients and their work context.

In addition to better understanding the work capacity, presenteeism, absenteeism, and early exit from the labor market, studies focusing on patients with rarer diseases, such as systemic sclerosis, primary Sjögren’s syndrome, and myositis, are essential to develop strategies to mitigate the burden of these conditions. Finally, to limit damage and restore functionality in Brazilian rheumatic patients, studies addressing the themes “return to work” and “reintegration into the labor market” are mandatory.

## References

[1] Scherlinger M, Mertz P, Sagez F, Meyer A, Felten R, Chatelus E, et al. Worldwide trends in all-cause mortality of auto-immune systemic diseases between 2001 and 2014. Autoimmun Rev. 2020;19(6):102531. 10.1016/j.autrev.2020.102531 32234406

[2] Mau W, Listing J, Huscher D, Zeidler H, Zink A. Employment across chronic inflammatory rheumatic diseases and comparison with the general population. J Rheumatol. 2005;32(4):721-8. 15801031

[3] Puolakka K, Kautiainen H, Möttönen T, Hannonen P, Hakala M, Korpela M, et al. Predictors of productivity loss in early rheumatoid arthritis: a 5 year follow up study. Ann Rheum Dis. 2005;64(1):130-3. 10.1136/ard.2003.019034 PMC1755184 15608311

[4] Allaire SH, Anderson JJ, Meenan RF. Reducing work disability associated with rheumatoid arthritis: identification of additional risk factors and persons likely to benefit from intervention. Arthritis Rheum. 1996;9(5):349-57. 10.1002/1529-0131(199610)9:5<349::aid-anr1790090503>3.0.co;2-g 8997924

[5] Roelen CAM, Norder G, Koopmans PC, Rhenen W, Klink JJL, Bültmann U. Employees sick-listed with mental disorders: who returns to work and when? J Occup Rehabil. 2012;22(3):409-17. 10.1007/s10926-012-9363-3 22447276

[6] Sabariego C, Coenen M, Ito E, Fheodoroff K, Scaratti C, Leonardi M, et al. Effectiveness of integration and re-integration into work strategies for persons with chronic conditions: a systematic review of European strategies. Int J Environ Res Public Health. 2018;15(3):552. 10.3390/ijerph15030552 PMC5877097 29562715

[7] Jorge LL, Gerard C, Revel M. Evidences of memory dysfunction and maladaptive coping in chronic low back pain and rheumatoid arthritis patients: challenges for rehabilitation. Eur J Phys Rehabil Med. 2009;45(4):469-77. 20032904

[8] Nicolau G, Yogui MM, Vallochi TL, Gianini RJ, Laurindo IMM, Novaes GS. Sources of discrepancy in patient and physician global assessments of rheumatoid arthritis disease activity. J Rheumatol. 2004;31(7):1293-6. 15229946

[9] Sacilotto NC, Giorgi RDN, Vargas-Santos AB, Albuquerque CP, Radominski SC, Pereira IA, et al. Real - rheumatoid arthritis in real life - study cohort: a sociodemographic profile of rheumatoid arthritis in Brazil. Adv Rheumatol. 2020;60:20. 10.1186/s42358-020-0121-5 32171331

[10] 0. Xavier RMH, Zerbini CAF, Pollak DF, Morales-Torres JLA, Chalem P, Restrepo JFM, et al. Burden of rheumatoid arthritis on patients’ work productivity and quality of life. Adv Rheumatol. 2019;59:47. 10.1186/s42358-019-0090-8 31706348

[11] Gomes RKS, Schreiner LC, Vieira MO, Machado PH, Nobre MRC. Staying in the labor force among patients with rheumatoid arthritis and associated factors in Southern Brazil. Adv Rheumatol. 2018;58(1):14. 10.1186/s42358-018-0009-9 30657075

[12] Pinheiro GRC, Khandker RK, Sato R, Rose A, Piercy J. Impact of rheumatoid arthritis on quality of life, work productivity and resource utilisation: an observational, cross-sectional study in Brazil. Clin Exp Rheumatol. 2013;31(3):334-40. 23324808

[13] Azevedo ABC, Ferraz MB, Ciconelli RM. Indirect costs of rheumatoid arthritis in Brazil. Value Health. 2008;11(5):869-77. 10.1111/j.1524-4733.2008.00332.x 18489511

[14] Louzada P, Souza BDB, Toledo RA, Ciconelli RM. [Descriptive analysis of the demographical and clinical characteristics of the patients with rheumatoid arthritis in the state of São Paulo, Brazil]. Rev Bras Reumatol. 2007;47(2):84-90. Portuguese. 10.1590/s0482-50042007000200002

[15] Teixeira RCA, Borba Neto EF, Christopoulos GB, Sato EI. The influence of income and formal education on damage in Brazilian patients with systemic lupus erythematosus. J Clin Rheumatol. 2017;23(5):246-51. 10.1097/RHU.0000000000000541 28700531

[16] Appenzeller S, Cendes F, Costallat LTL. Cognitive impairment and employment status in systemic lupus erythematosus: a prospective longitudinal study. Arthritis Rheum. 2009;61(5):680-7. 10.1002/art.24346 19405004

[17] Koopman C, Pelletier KR, Murray JF, Sharda CE, Berger ML, Turpin RS, et al. Stanford Presenteeism Scale: health status and employee productivity. J Occup Environ Med. 2002;44(1):14-20. 10.1097/00043764-200201000-00004 11802460

[18] Leggett S, Zee-Neuen A, Boonen A, Beaton DE, Bojinca M, Bosworth A, et al. Test-retest reliability and correlations of 5 global measures addressing at-work productivity loss in patients with rheumatic diseases. J Rheumatol. 2016;43(2):433-9. 10.3899/jrheum.141605 26628608

[19] Gignac MAM, Badley EM, Lacaille D, Cott CC, Adam P, Anis AH. Managing arthritis and employment: making arthritis-related work changes as a means of adaptation. Arthritis Rheum. 2004;51(6):909-16. 10.1002/art.20822 15593110

[20] 0. Lerner D, Amick BC 3rd, Rogers WH, Malspeis S, Bungay K, Cynn D. The work limitations questionnaire. Med Care. 2001;39(1):72-85. 10.1097/00005650-200101000-00009 11176545

[21] Reilly MC, Zbrozek AS, Dukes EM. The validity and reproducibility of a work productivity and activity impairment instrument. Pharmacoeconomics. 1993;4(5):353-65. 10.2165/00019053-199304050-00006 10146874

[22] Tang K, Beaton DE, Boonen A, Gignac MAM, Bombardier C. Measures of work disability and productivity: Rheumatoid Arthritis Specific Work Productivity Survey (WPS-RA), Workplace Activity Limitations Scale (WALS), Work Instability Scale for Rheumatoid Arthritis (RA-WIS), Work Limitations Questionnaire (WLQ), and Work Productivity and Activity Impairment Questionnaire (WPAI). Arthritis Care Res (Hoboken). 2011;63 Suppl 11:S337-49. 10.1002/acr.20633 22588755

